# Chronic Exercise Improves Mitochondrial Function and Insulin Sensitivity in Brown Adipose Tissue

**DOI:** 10.3389/fphys.2018.01122

**Published:** 2018-08-17

**Authors:** Natalia de las Heras, Mercedes Klett-Mingo, Sandra Ballesteros, Beatriz Martín-Fernández, Óscar Escribano, Javier Blanco-Rivero, Gloria Balfagón, Marta L. Hribal, Manuel Benito, Vicente Lahera, Almudena Gómez-Hernández

**Affiliations:** ^1^Department of Physiology, School of Medicine, Complutense University of Madrid, Madrid, Spain; ^2^Department of Biochemistry and Molecular Biology II, School of Pharmacy, Complutense University of Madrid, Madrid, Spain; ^3^CIBER of Diabetes and Associated Metabolic Diseases, Barcelona, Spain; ^4^Department of Physiology, School of Medicine, Autonomous University of Madrid, Madrid, Spain; ^5^Department of Medical and Surgical Sciences, Magna Græcia University of Catanzaro, Catanzaro, Italy

**Keywords:** mitochondrial dynamic, insulin sensitivity, exercise, brown adipose tissue, UCPs

## Abstract

The aim of the present work was to study the consequences of chronic exercise training on factors involved in the regulation of mitochondrial remodeling and biogenesis, as well as the ability to produce energy and improve insulin sensitivity and glucose uptake in rat brown adipose tissue (BAT). Male Wistar rats were divided into two groups: (1) control group (C; *n* = 10) and (2) exercise-trained rats (ET; *n* = 10) for 8 weeks on a motor treadmill (five times per week for 50 min). Exercise training reduced body weight, plasma insulin, and oxidized LDL concentrations. Protein expression of ATP-independent metalloprotease (OMA1), short optic atrophy 1 (S-OPA1), and dynamin-related protein 1 (DRP1) in BAT increased in trained rats, and long optic atrophy 1 (L-OPA1) and mitofusin 1 (MFN1) expression decreased. BAT expression of nuclear respiratory factor type 1 (NRF1) and mitochondrial transcription factor A (TFAM), the main factors involved in mitochondrial biogenesis, was higher in trained rats compared to controls. Exercise training increased protein expression of sirtuin 1 (SIRT1), peroxisome proliferator-activated receptor γ coactivator 1α (PGC1α) and AMP-activated protein kinase (pAMPK/AMPK ratio) in BAT. In addition, training increased carnitine palmitoyltransferase II (CPT II), mitochondrial F1 ATP synthase α-chain, mitochondrial malate dehydrogenase 2 (mMDH) and uncoupling protein (UCP) 1,2,3 expression in BAT. Moreover, exercise increased insulin receptor (IR) ratio (IRA/IRB ratio), IRA-insulin-like growth factor 1 receptor (IGF-1R) hybrids and p42/44 activation, and decreased IGF-1R expression and IR substrate 1 (p-IRS-1) (S307) indicating higher insulin sensitivity and favoring glucose uptake in BAT in response to chronic exercise training. In summary, the present study indicates that chronic exercise is able to improve the energetic profile of BAT in terms of increased mitochondrial function and insulin sensitivity.

## Introduction

Brown adipose tissue (BAT) plays a major role in body energy expenditure and contains numerous mitochondria that function to mediate adaptive thermogenesis and protect against hypothermia and obesity (Cannon and Nedergaard, 2004). The activation of BAT offers a new way to battle obesity and other metabolic disorders, closely associated with both mitochondrial function alterations and reduced mitochondrial density (Patti et al., 2003; Morino et al., 2005).

Mitochondria are dynamic organelles that continuously are subjected to dynamic processes: (a) the fusion of two mitochondria in a single; (b) the fission of mitochondria into smaller ones; and (c) the biogenesis, required for cell growth and adaptation to increased oxidative stress and nutritional deprivation. Mitochondrial dynamics is a highly regulated process that controls mitochondrial density in the cells and may be changed depending on the physiological cell state. In mammals, mitochondrial fusion is mediated by mitofusin 1 and 2 (Mfn1 and Mfn2) and the dynamic-related GTPase, optic atrophy-1 (OPA1), which are located in the outer and inner membranes, respectively (Malka et al., 2005; Quiros et al., 2012). Mitochondrial fission is mediated by dynamin-related protein 1 (Drp1), which is mainly located in the cytosol (Smirnova et al., 2001). Mitochondria go through continuous cycles of selective fusion and fission, referred to as the “mitochondrial life cycle,” to maintain the quality of its function. Deregulation of fusion/fission events appears to be involved in several diseases. The functions of OPA1 are controlled by alternative splicing and proteolysis of different isoforms, long and short isoforms (L-OPA1 and S-OPA1) (Head et al., 2009). The ATP-independent metalloprotease OMA1, a proteolytic enzyme located in the inner mitochondrial membrane, is responsible for OPA1 proteolysis (Quiros et al., 2013). Stress conditions, such as the induction of apoptosis and the specific dissipation of mitochondrial membrane potential, induce the cleavage of OPA1 to short isoforms by OMA1, resulting in the inhibition of mitochondrial fusion (Head et al., 2009; Jiang et al., 2014). Peroxisome proliferator-activated receptor-γ coactivator 1α (PGC1α) is a major regulator of mitochondrial biogenesis by activating different transcription factors, including the nuclear respiratory factor type 1 (Nrf1). Nrf1 interacts with mitochondrial transcription factor A (Tfam), which drives transcription and replication of mitochondrial DNA and, therefore, mitochondrial biogenesis (Vina et al., 2009). Both AMP-activated protein kinase (AMPK) and the silent information regulator protein (Sir2) homolog SIRT1 are activators of PGC1α (Fulco and Sartorelli, 2008).

The insulin receptor (IR) is a member of the tyrosine kinase receptor superfamily with an essential role in glucose metabolism (Benyoucef et al., 2007; Ward and Lawrence, 2009). The IR is closely related to other receptors such as the IGF type I receptor (IGF-IR) that is involved in normal growth and development (Ward and Lawrence, 2009). In mammals, alternative splicing gives rise to two isoforms of IR: IRA and IRB. IRB has 12 additional amino acids encoded by exon 11 (Whittaker et al., 2002). This sequence is located immediately downstream of the ligand binding domain but does not affect insulin binding affinity (Whittaker et al., 2002; Menting et al., 2013). Moreover, IRA is predominantly expressed during fetal development enhancing the effects of IGF-II (Frasca et al., 1999). Conversely, IRB is the predominant IR in adult tissues, including the liver, where it triggers the metabolic effects of insulin (Malaguarnera et al., 2011).

Exercise elicits changes and adaptations in the energy metabolism in skeletal muscle, liver, as well as in white and BAT (Laye et al., 2009; Slocum et al., 2013; Drake et al., 2016). One of the most profound effects of exercise training is an increase in mitochondrial content in skeletal muscle, since higher mitochondrial content will increase fat utilization and reduce the formation of lactic acid and other fatigue related substances at a given submaximal workload (Stisen et al., 2006). In white adipose tissue from healthy non-obese rats, exercise training increased the expression of several proteins involved in mitochondrial biogenesis regulation, such as PGC1α and TFAM (Sutherland et al., 2009). However, information on the effects of chronic exercise on mitochondrial dynamics in BAT is scarce. Moreover, uncoupling proteins (UCPs) play a pivotal role in the metabolic adaptation of BAT to exercise training, increasing the oxidation of metabolic substrates necessary for sustaining enhanced thermogenesis in order to produce heat (Boss et al., 1998; Ricquier, 2006). On the other hand, it has been described that chronic exercise improves insulin sensitivity (Wagner et al., 2016; Jimenez-Maldonado et al., 2017), favoring glucose uptake in the muscle (Kim, 2016), controlling glucose tolerance (Molsted et al., 2013), as well as preventing vascular dysfunction (Lee et al., 2011). However, there is nothing described whether exercise might improve insulin sensitivity in BAT and what mechanisms might be involved in such improvement.

Therefore, the aim of the present work was to study the consequences of chronic exercise training on factors involved in the regulation of mitochondrial dynamic and the ability to produce energy and improve insulin sensitivity and glucose uptake in rat BAT. For this purpose, protein expression levels of factors involved in mitochondrial remodeling (OMA1, OPA1, Mfn1, and Drp1), mitochondrial biogenesis (NRF1 and TFAM), and main factors from PGC1α signaling cascades as well as proteins implicated in insulin signaling pathway (IR, IRA, IRB, IGF-1R, p-ERK, p-AKT, p-IRS-1) were investigated in interscapular BAT from rats subjected to chronic exercise training. In addition, to evaluate mitochondrial proteins involved in energy/heat production, the expression levels of carnitine palmitoyltransferase II (CPTII), mitochondrial malate dehydrogenase 2 (mMDH), mitochondrial F1 ATP synthase α-chain and UCP1,2,3 were also determined in rat interscapular BAT.

## Materials and Methods

### Animals

Three-month-old male Wistar rats (initial weight: 334.6 ± 3.9 g) were obtained from the Animal Quarters of the Autonoma University of Madrid (Registration number EX-021U; ES-280790000097). This study was carried out in accordance with the recommendations of the European Union guidelines on the ethical care of experimental animals (DG XI of the European Commission, Directive 2010/63/EU, of September 22, 2010, Annex IV). The protocol was approved by the Ethics Committee of the Autonoma University of Madrid (CEI-38-839; RD 1201/2005). Rats were housed at a constant room temperature, humidity, and light cycle (12:12-h light-dark) and had free access to tap water and standard rat chow (#2014, Harlan Teklad, MN, United States) *ad libitum*. Animals were weighed every 2 weeks. Rats were divided into two experimental groups: (1) control group (C; *n* = 10) and (2) rats trained for 8 weeks (ET; *n* = 10). Rats were fasting overnight for 12 h before euthanasia. After being sacrificed the muscle soleus and the interscapular BAT pads were removed, weighed, and washed in saline solution, dried and immediately frozen and stored at -80°C.

### Exercise Training Protocol and Citrate Synthase Activity

Exercise training was performed on a motor treadmill (Motor-driven Treadmill LI8706, Letica Scientific Instruments, Barcelona, Spain) for 8 weeks, five times per week for 50 min each session, gradually progressing toward 55–65% of maximal running speed (15–20 m/min), as described elsewhere (Graham and Rush, 2004; Blanco-Rivero et al., 2013). To determine the maximal exercise capacity, rats were subjected to a progressive exercise test on a treadmill using an incremental speed protocol of 5 m/min every 3 min and no grade until exhaustion. The treadmill exercise test was repeated after 5 weeks of exercise training in order to adjust training intensity. Rats were considered to be exhausted when they could no longer run at the treadmill speed (Blanco-Rivero et al., 2013). The sedentary or control rats were handled at least twice a week for habituation to the experimental protocols.

Citrate synthase activity was used as a marker of muscle oxidative activity (Alp et al., 1976). The enzyme activity was measured in whole muscle homogenates, and the complex resulting from acetyl-CoA and oxaloacetate was determined at 412 nm (ASYS UVM 340, Biochrom, Cambridge, United Kingdom) and 25°C, at an interval of 10 min. Citrate synthase activity was expressed as nmol/min per mg of protein.

### Metabolic Parameters

Plasma concentrations of glucose, total cholesterol, and triglycerides were determined using spectrophotometric techniques in an autoanalyzer (Vitros Fusion 5,1, Diagnostics Ortho Clinical, Johnson & Johnson, New Brunswick, NJ, United States).

Plasma concentration of insulin and oxidized LDL were measured with specific quantitative sandwich enzyme immunoassay (EZRMI-13K, Millipore-Bedford, Burlington, MA, United States and Biomedica Medizinprodukte GmbH & Co., KG, Vienna, Austria, respectively). Absorbance results were read on a spectrophotometer at a wavelength of 450 nm (Reader UVM 340, Asys Hitech GmbH, Austria).

### Western Blot Analysis

Interscapular BAT samples (100 mg) were homogenized in 300 μL of lysis buffer (ReadyPreTM 2-D Starter Kit Rehydration/Sample Buffer #1632106; Bio-Rad Laboratories, Inc., Hercules, CA, United States) mixed with protease inhibitor cocktail (Roche Applied Science, Mannheim, Germany) and phenylmethylsulfonyl fluoride (Sigma-Aldrich, Co., Spain) with the automatic homogenizer Bullet Blender, following the manufacturer’s instructions (Cultek, SSB14B, Next Advance, Inc., United States). After homogenization, samples were kept on ice for 30 min, shaken briefly and centrifuged at 10,000 *g* for 10 min at 4°C. Finally, proteins were collected from supernatant. Samples were separated by sodium dodecyl sulfate-polyacrylamide gel electrophoresis under reducing conditions. After electrophoresis, samples were transferred to polyvinylidene difluoride membranes (Millipore-Bedford, Burlington, MA, United States). The membranes were blocked in PBS containing 0.1% Tween-20 and 5% dry skimmed milk for 1 h at room temperature, and were then incubated in the same buffer with specific antibodies for 18 h at 4°C. OPA1 [1:2000; 612607] (BD Biosciences, Madrid, Spain); OMA1 [1:500; ab104316], Mfn1 [1:1000; ab57602], Drp1 [1:500; ab56788], AMPK [1:1000; ab80039], phospho AMPK (pAMPK) [1:1000; ab72845], SIRT1 [1:500; ab110304], PGC1α [1:250; ab106814], uncoupling protein 1 (UCP-1) [1:1000; ab23841], UCP-2 [1:500; ab67241], UCP-3 [1:1000; ab3477], NRF1 [1:1000; ab175932], and TFAM [1:1000; ab131607] (Abcam, Cambridge, United Kingdom); mitochondrial F1 ATP synthase α-chain [1:1000; sc-58613], carnitine palmitoyltransferase II (CPTII) [1:1000; sc-20526], mitochondrial malate dehydrogenase 2 (mMDH) [1:1000; sc133777], IR [1:1000; sc-711] and insulin-like growth factor 1 receptor (IGF-1R) [1:1000; sc-713] (Santa Cruz Biotechnology, Dallas, TX, United States); p-IRS-1 (S307) [1:1000; 13110], and p-p42/44 (Thr202/Tyr204) [1:1000; 9102] (Cell Signaling, St. Louis, MO, United States). The anti-IRB antibody (plus exon 11) was kindly provided by Dr. Sesti and Dr. Hribal. After washing, detection was made through incubation with peroxidase-conjugated secondary antibody, and developed using an ECL chemiluminescence kit (Millipore-Bedford, Burlington, MA, United States). As loading control we used β-actin [1:10000] (Sigma Aldrich, Co., Spain). In addition, Red Ponceau staining was used to show the quality of proteins and efficacy of protein transfer to the membrane (not shown). The detection for blots was made using the GeneGnome5 (Syngene Bio Imaging; Synoptics Ltd., Cambridge, United Kingdom) obtaining a chemiluminescence imaging. Results are expressed as an n-fold increase over the values of the control group in densitometric arbitrary units.

### Immunoprecipitation

A total of 150 μg protein extracts from BAT was immunoprecipitated with IRB isoform antibody. The supernatants from the first immunoprecipitation were immunoprecipitated with IRβ antibody (recognizes two IR isoforms). Thus, immune complexes (only IRA) were collected on protein A-agarose beads and submitted to SDS–PAGE. Finally, the immunoblots were incubated with anti-IGF-1R antibodies to study the association between IRA or IRB isoform and IGF-1R.

### Statistical Analysis

All analyses and graphs were performed using GraphPad Prism 5 (GraphPad Software). Data were compared by the Lilliefors test followed by Student’s *t*-test. Values are presented as means ± standard error of mean (SEM). The level of significance was set at *p* < 0.05.

## Results

### General and Metabolic Characteristics

As expected, exercise training reduced (*p* < 0.05) body weight and body weight gain in rats. Exercise training decreased (*p* < 0.05) basal plasma insulin levels without modifying glucose levels. Plasma total cholesterol and triglyceride levels were similar in both control and trained rats. Chronic exercise training decreased (*p* < 0.05) plasma oxidize LDL concentration. Citrate synthase activity in soleus was higher (*p* < 0.05) in trained rats compared to control animals (**Table 1**).

**Table 1:** General characteristics and metabolic systemic parameters.

	C	ET
Body weight (g)	429 ± 6.4	394 ± 5.5^*^
Increased weight (g)	91.1 ± 5.2	66.4 ± 4.6^*^
Glucose (mg/dl)	102.5 ± 2.4	103.4 ± 2.7
Insulin (ng/ml)	0.42 ± 0.02	0.35 ± 0.01^*^
Total cholesterol (mg/dl)	65.2 ± 0.9	63.6 ± 2.3
Triglycerides (mg/dl)	59.3 ± 2.1	58.7 ± 2.1
Oxidize LDL (μg/dl)	52.1 ± 7.9	37.3 ± 2.7^*^
Citrate synthase (nmol/min mg protein)	57.3 ± 2.6	72.3 ± 1.3^*^


*Final body and increased weight; plasma concentrations of glucose, insulin, total cholesterol, triglycerides, and oxidize LDL; and citrate synthase activity in soleus in control rats (C) and exercise trained rats (ET) for 8 weeks. Data are expressed as mean ± SEM. (*n* = 10 animals per group). ^∗^*p* < 0.05 vs. C.*

### Expression of Factors Involved in Mitochondrial Remodeling

**FIGURE 1:**
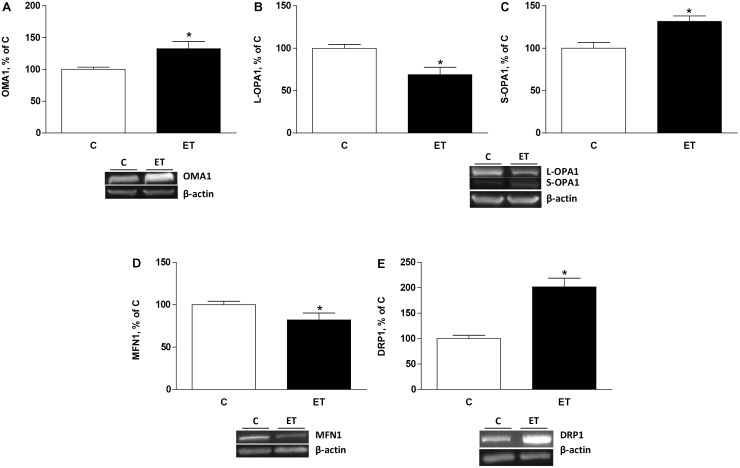
Quantitative analyses of protein levels by western blot for **(A)** ATP-independent metalloprotease OMA1, **(B)** long optic atrophy 1 (L-OPA1), **(C)** short optic atrophy 1 (S-OPA1), **(D)** mitofusin 1 (Mfn1), and **(E)** dynamin related protein 1 (Drp1) in brown adipose tissue of control rats (C) and trained rats (ET) for 8 weeks. Data are expressed as mean ± SEM. (*n* = 10 animals per group). ^∗^*p* < 0.05 vs. C.

To evaluate the effects of exercise training on mitochondrial remodeling, OMA1, OPA1, Mfn1, and Drp1 were evaluated. Exercise training increased (*p* < 0.05) protein expression of OMA1 and S-OPA1 in BAT, and expression of L-OPA1 decreased (*p* < 0.05) in rats under chronic exercise compared to control (**Figures 1A–C**). Mfn1 protein expression decreased (*p* < 0.05) in BAT of trained rats compared to control rats (**Figure 1D**). Furthermore, chronic exercise training increased (*p* < 0.05) protein expression of Drp1 expression in BAT (**Figure 1E**).

### Expression of Factors Involved in Mitochondrial Biogenesis

**FIGURE 2:**
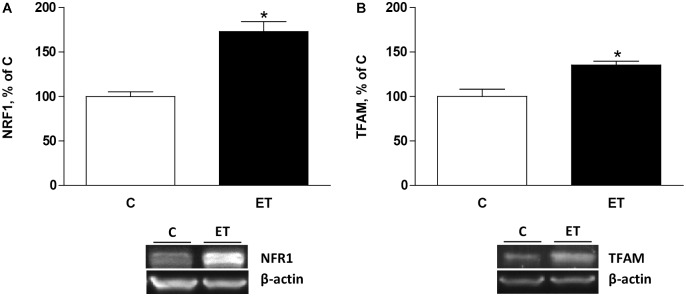
Quantitative analyses of protein levels by western blot for **(A)** nuclear respiratory factor type 1 (NRF1) and **(B)** mitochondrial transcription factor A (TFAM) in brown adipose tissue of control rats (C) and trained rats (ET) for 8 weeks. Data are expressed as mean ± SEM. (*n* = 10 animals per group). ^∗^*p* < 0.05 vs. C.

To evaluate the effects of exercise training on mitochondrial biogenesis, NRF1 and TFAM were evaluated. Chronic exercise training increased (*p* < 0.05) protein expression of NRF1 and TFAM protein expression in BAT, suggesting an enhancement of mitochondrial biogenesis (**Figures 2A,B**).

### Expression of Factors From PGC1α Signaling Cascade

**FIGURE 3:**
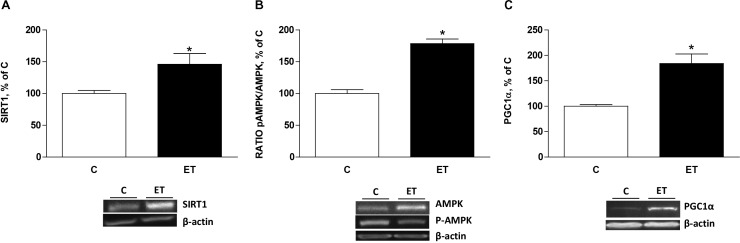
Quantitative analyses of protein levels by western blot for **(A)** sirtuin 1 (SIRT1), **(B)** pAMPK/AMPK ratio and **(C)** peroxisome proliferator-activated receptor γ coactivator 1α (PGC1α) in brown adipose tissue of control rats (C) and trained rats (ET) for 8 weeks. Data are expressed as mean ± SEM. (*n* = 10 animals per group). ^∗^*p* < 0.05 vs. C.

Protein expression of SIRT1, ratio pAMPK/AMPK and PGC1α were measured to evaluate factors regulating mitochondrial biogenesis. Protein expression of SIRT1 and ratio pAMPK/AMPK in BAT was higher (*p* < 0.05) in trained rats compared to control rats (**Figures 3A,B**). It was accompanied of increased expression (*p* < 0.05) of PGC1α in trained rats (**Figure 3C**).

### Mitochondrial Proteins Involved in Energy and Heat Production

**FIGURE 4:**
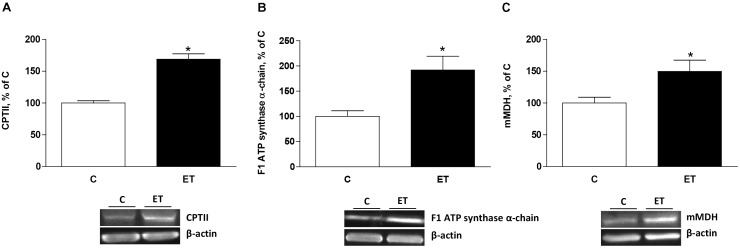
Quantitative analyses of protein levels by western blot for **(A)** carnitine palmitoyltransferase II (CPTII), **(B)** mitochondrial F1 ATP synthase α-chain ATP synthase and **(C)** mitochondrial malate dehydrogenase 2 (mMDH) in brown adipose tissue of control rats (C) and trained rats (ET) for 8 weeks. Data are expressed as mean ± SEM. (*n* = 10 animals per group). ^∗^*p* < 0.05 vs. C.

**FIGURE 5:**
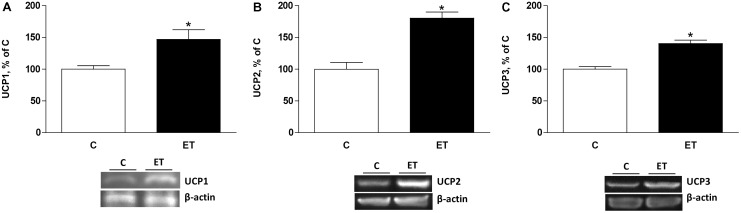
Quantitative analyses of protein levels by western blot for **(A)** uncoupling protein 1 (UCP1), **(B)** UCP2, and **(C)** UCP3 in brown adipose tissue of control rats (C) and trained rats (ET) for 8 weeks. Data are expressed as mean ± SEM. (*n* = 10 animals per group). ^∗^*p* < 0.05 vs. C.

Exercise training increased (*p* < 0.05) CPTII, mitochondrial F1 ATP synthase α-chain expression and mMDH (**Figures 4A–C**) in BAT. UCP1, UCP2, and UCP3 expression increased (*p* < 0.05) in BAT from rats under chronic exercise compared to controls (**Figures 5A–C**).

### Differential IR, IGF-IR, and Hybrid Receptors Induced by Exercise

**FIGURE 6:**
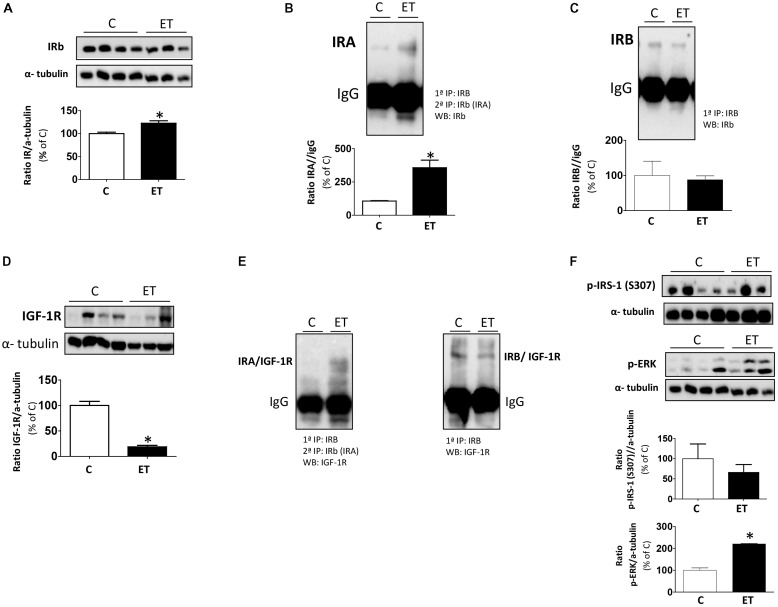
Insulin signaling in BAT from control rats (C) and trained rats (ET). Effect of chronic exercise in protein levels of **(A)** IR, **(B)** IRA, **(C)** IRB, **(D)** IGF-1R, **(E)** Hybrid receptors: IRA/IGF-1R or IRB/IGF-1R and **(F)** p-IRS-1 (S307) and p-p42/44 by Western blot. To analyze the specific isoforms IR, we first performed immunoprecipitations against IRB antibody and after immunoprecipitations against IRβ. Data are expressed as mean ± SEM. (*n* = 10 animals per group). ^∗^*p* < 0.05 vs. C. The loading control, α-tubulin, of panels **(A,F)** is the same because both Western blots against IRβ and p-ERK antibodies were performed in the same membrane.

Exercise training increased (*p* < 0.05) the expression of total IR as compared to controls (**Figure 6A**). Regarding IR isoforms, exercise increased (*p* < 0.05) the expression of IRA while it did not change IRB levels (**Figures 6B,C**). However, the expression of IGF-1R was dramatically decreased (*p* < 0.05) in rats under chronic exercise (**Figure 6D**). Next, we measured the association between IGF-1R and IR isoforms (**Figure 6E**), and the results revealed that chronic exercise induced an increased formation of IRA/IGF-1R hybrid receptors as compared to IRB/IGF-1R hybrids. Finally, we evaluated the insulin sensitivity in control and exercise training groups by means of ERK and S307-IRS-1 phosphorylation. Our results showed that chronic exercise improves insulin sensitivity increasing ERK phosphorylation and decreasing S307-IRS-1 phosphorylation (**Figure 6F**). The loading control, α-tubulin, of **Figures 6A,F** is the same because both Western blots against IRβ and p-ERK antibodies were performed in the same membrane (**Supplementary Presentation 1**).

## Discussion

The present study describes for the first time, that exercise training in rats modifies mitochondrial dynamics in BAT. Enhanced expression of factors regulating mitochondrial biogenesis and mitochondrial fission, together with decreased expression of factors regulating mitochondrial fusion were produced. These effects occurred together with enhanced expression of UCP1, UCP2, and UCP3 which suggests increased heat production, as a possible metabolic adaptation of BAT to chronic exercise training. Moreover, an increase of IRA/IRB ratio, IRA-IGF-1R hybrids, and p42/44 activation as well as a significant decrease of IGF-1R expression and p-IRS-1 (S307) indicate higher insulin sensitivity and favored glucose uptake in BAT in response to chronic exercise training.

Exercise training has long been known to promote mitochondrial biogenesis in skeletal muscle. However, the effects of chronic exercise training on mitochondrial dynamics in BAT are fairly unknown. The present study shows that chronic exercise training increased pAMPK/AMPK ratio and the expression of SIRT1 in BAT, which could be responsible for the observed enhanced expression of PGC1α. Indeed, SIRT1 deacetylates PGC1α while AMPK phosphorylates PGC1α, resulting in promotion of mitochondrial biogenesis (Dominy et al., 2010). Mitochondrial biogenesis depends upon the activity of nuclear transcription factors including NRF1 and TFAM (Virbasius and Scarpulla, 1994). Thus it could be proposed that under the present conditions, enhancement of PGC1α would activate NRF1 promoting the expression of TFAM, and consequently mitochondrial differentiation and biogenesis.

Fusion/fission equilibrium is a key element for mitochondrial growth and redistribution, and for maintenance of a quality mitochondrial network (Song et al., 2007; Head et al., 2009; Anand et al., 2014). Several human (Cartoni et al., 2005; Fealy et al., 2014) and experimental (Ding et al., 2010; Caffin et al., 2013) studies on mitochondrial fusion/fission equilibrium have been conducted in skeletal muscle during exercise, although mitochondrial remodeling in BAT in response to chronic exercise training is fairly unknown. The present study shows that exercise training increased OMA1 and S-OPA1 expression together with a decrease in L-OPA1. These results together with the observed reduction of Mfn1 expression indicates reduced mitochondrial fusion under the present experimental circumstances. Previous results also showed that chronic exercise training could be considered a stressful condition, increasing both OMA1 expression and cleavage of L-OPA1, which leads to an increased expression of S-OPA1 (Quiros et al., 2012). The proteolysis of OPA1 by OMA1 leads to the formation of several subunits of OPA1 by alternative splicing, generating fusion-incompetent OPA1 and cessation of inner membrane fusion (Malka et al., 2005). This situation is further supported by the observed increased expression of Drp1, a protein involved in mitochondrial fission. Therefore, it could be proposed that changes in mitochondrial biogenesis and fusion/fission equilibrium, could be considered as part of the metabolic adaptation of BAT to chronic exercise, in order to maintain overall mitochondrial quality.

Mitochondrial content and respiratory capacity can be modified according to specific metabolic conditions such as exercise (Liesa and Shirihai, 2013). Under standard conditions, mitochondrial respiration is coupled to ATP production and constitutes the main source of ATP. However, as the coupling of respiration to ADP phosphorylation is less than 100% efficient energetically, respiration also releases heat (Ricquier, 2006). UCPs such as UCP1 are mitochondrial proteins able to dissipate the proton gradient generated by NADH-powered pumping of protons from the mitochondrial matrix to the mitochondrial intermembrane space. The energy lost in dissipating the proton gradient by UCP action is not used to produce ATP but to generate heat. Thus, UCP1 plays a key role in BAT physiology since it confers to brown adipocytes their specific capacity to dissipate oxidation energy as heat (Ricquier et al., 1986; Ricquier, 2006). As previously published, our study shows an increased expression of UCP1 indicating that exercise training increases UCP1 in order to produce heat (Boss et al., 1998). In addition, the results also show increased expression of UCP2 and UCP3 in BAT, probably as a co-operative response to chronic exercise training to enhance heat production. To our knowledge, few studies revealed increased expression of UCP2 and three during chronic exercise in BAT. PGC-1α, as a master regulator of mitochondrial biogenesis and oxidative metabolism, is highly expressed in tissues with high oxidative metabolism such as BAT and skeletal muscle (Knutti and Kralli, 2001; Puigserver and Spiegelman, 2003), and in our study would participate actively in the activation of UCP1. Similarly, it is known that is strongly induced in BAT by cold exposure, and in turn, stimulates the adaptive thermogenic program by inducing UCP1 gene (Ricquier et al., 1986; Puigserver et al., 1998). This is achieved by the numerous mitochondria in brown adipocytes and UCPs, associated with increased biogenesis and mitochondrial fission, which activates respiration and diverts oxidation-free energy to thermogenesis (Ricquier, 2006). In addition, several studies show that the increase in mitochondrial fission potentiates free fatty acid-induced uncoupling and enables the tissue to be more thermogenic (Wikstrom et al., 2014).

One of the consequences could be an increased mitochondrial capacity to generate ATP, would match energy demands during chronic exercise. This notion is supported by the observed increase of CPTII, mMDH, and ATP synthase, which promote fatty acid transport and beta-oxidation into the mitochondria, Krebs’s cycle and oxidative phosphorylation, respectively. On the other hand, an increase in uncoupling is common features of various conditions that lead to mitochondrial fragmentation (Parone et al., 2008; Wikstrom et al., 2014). This is further supported by the observed increased expression of Drp1 and S-OPA in BAT of rats subjected to chronic physical exercise. These results provide evidences that mitochondrial fission is not deleterious *per se* but it regulates uncoupled respiration and thus increases energy expenditure by promoting shifting nutrient oxidation toward heat production, rather than toward mitochondrial ATP synthesis (Twig and Shirihai, 2011).

Finally, it is widely known that physical exercise is able to ameliorate insulin sensitivity in myocytes by increasing glucose uptake (Birnbaum, 1989; James et al., 1989; Neufer and Dohm, 1993; Gurley et al., 2016). In this sense, we demonstrate that chronic exercise increases IR expression in BAT, moreover, we observe a decrease in IRS-1 (S307) phosphorylation as well as an increased p42/p44 phosphorylation suggesting that chronic exercise is also able to increase insulin sensitivity in BAT. In addition, it has been shown that the IRA/IRB ratio can be modified in several pathophysiological conditions (Nevado et al., 2006; Escribano et al., 2009), and our results show that chronic exercise training induces an increased IRA/IRB ratio in BAT. IRA has been described as a glucose uptake modulator in hepatocytes, vascular smooth muscle cells and beta cells (Nevado et al., 2006; Gomez-Hernandez et al., 2013; Escribano et al., 2015; Diaz-Castroverde et al., 2016a,b). Thus, the increased IRA/IRB ratio observed in chronic exercise trained rats could be also favoring glucose uptake in the BAT favoring glucose disposal and oxidation.

In summary, the present study indicates that chronic exercise is able to improve the energetic profile of BAT in terms of increased mitochondrial function and insulin sensitivity. Our results demonstrate a higher oxidative capacity of BAT that could help to improve the metabolic rate of the whole body in order to fight against lipid accumulation and obesity. A better understanding in the mechanisms involved could help to find new targets for obesity treatment.

## Author Contributions

NdlH and AG-H conceived and designed the experiments, performed the experiments, analyzed the data, and wrote the manuscript. MK-M and SB performed the experiments and analyzed the data. BM-F, JB-R, GB, and MB conceived and designed the experiments and analyzed the data. MH provided anti-IRB antibody (plus exon 11). VL conceived and designed the experiments, analyzed the data, and wrote the manuscript. All authors read and approved the final version of the manuscript.

## Conflict of Interest Statement

The authors declare that the research was conducted in the absence of any commercial or financial relationships that could be construed as a potential conflict of interest.
