# Supplementary material for: Chronic Exercise Improves Mitochondrial Function and Insulin Sensitivity in Brown Adipose Tissue
Source: Front Physiol. 2018 Aug 17;9:1122. doi: 10.3389/fphys.2018.01122 (PMC6107710; doi:10.3389/fphys.2018.01122)
Supplement: Supplementary file 1 [file Presentation_1.pdf]

The same membranes (1 and 2) were cut around 70 kDa (→). The upper part of two membranes were incubated with IRβ antibody (SC-711), obtaining a band of 95kDa corresponding to total IR (Figure 6A). The lower part of both membranes were firstly incubated with p-ERK antibody, obtaining two bands (42 and 44 kDa) (Figure 6F). After, two membranes were stripped and incubated with α-tubulin antibody (52 kDa) (Figure 6A and F). In both membranes, there are 14 samples of BAT. The first seven lanes correspond to BAT from rats under STD and the last seven lanes correspond to BAT from obese rats (data not included in this paper). I have indicated with the red box the images that we have included in this paper. Within the standard group (7 first lanes of each membrane), the first four lanes correspond to controls (C) and the next three correspond to trained rats (ET). The same membranes were incubated with three different antibodies (IRβ, p-ERK and α-tubulin). For this reason, α-tubulin from Figure 6A and F is the same. Moreover, you can see in the images the cutting marks demonstrate that they are the same membrane (*).

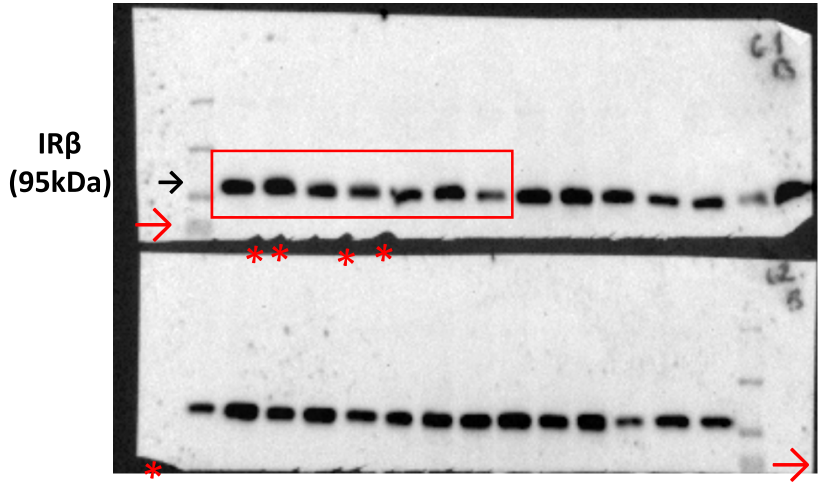

Membrane 1 and 2. WB Irβ (IRtotal) (Figure 6A)

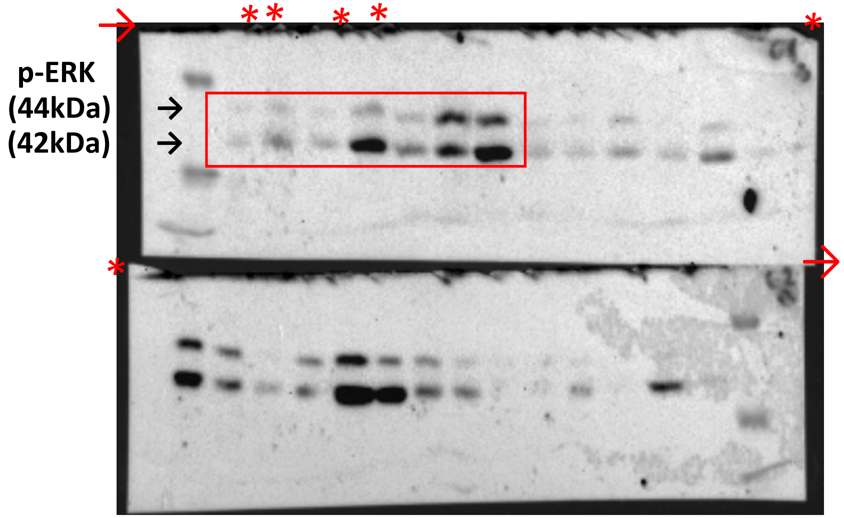

Membrane 1 and 2. WB p-ERK (Figure 6F)

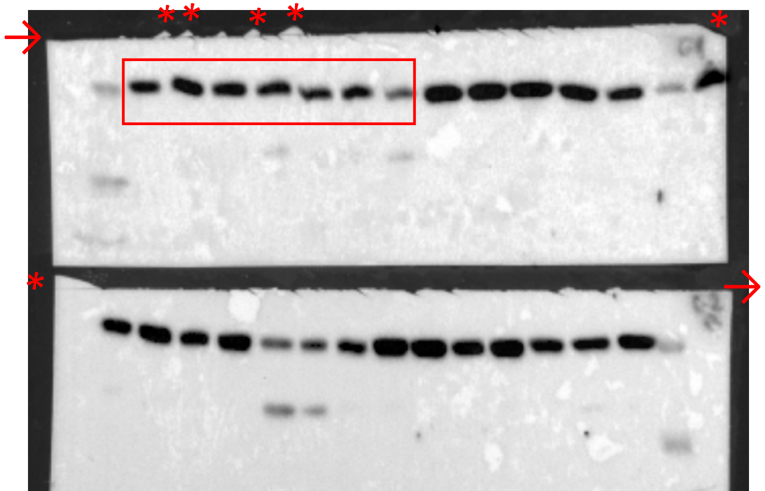

Membrane 1 and 2. WB α-tubulin (Figure 6A and F)
